# Advancing chronic pain care in Canada: History and impact of the Canadian Pain Task Force

**DOI:** 10.1080/24740527.2024.2358332

**Published:** 2024-06-10

**Authors:** Fiona Campbell, Manon Choinière, Hani El-Gabalawy, Jacques Laliberté, Michael Sangster, Jaris Swidrovich, Linda Wilhelm, Maria Hudspith

**Affiliations:** a Department of Anesthesia & Pain Medicine, Hospital for Sick Children, Toronto, Ontario, Canada; b Temerty Faculty of Medicine, University of Toronto, Toronto, Ontario, Canada; c Department of Anesthesiology and Pain Medicine, Faculty of Medicine, Université de Montréal, Montréal, Québec, Canada; d Research Center, Centre Hospitalier de l’Université de Montréal, Montréal, Québec, Canada; e Arthritis Centre and Manitoba Centre for Proteomics and Systems Biology, Rady Faculty of Health Sciences, University of Manitoba, Winnipeg, Manitoba, Canada; f Association Québécoise de la douleur chronique, Montréal, Québec, Canada; g Ambulatory Medical Care, IWK Health Centre, Halifax, Nova Scotia, Canada; h Canadian Physiotherapy Association, Ottawa, Ontario, Canada; i Leslie Dan Faculty of Pharmacy, University of Toronto, Toronto, Ontario; j Canadian Arthritis Patient Alliance, New Brunswick, Canada; k Pain BC, Vancouver, British Columbia, Canada

**Keywords:** chronic pain, Canada, Canadian Pain Task Force

## Abstract

In 2019, Health Canada established the Canadian Pain Task Force. Through this commitment, Canada joined other countries, such as the United States and Australia, in creating a national-level mechanism to support work in the area of chronic pain. This article provides a historical narrative of national and regional advocacy and efforts that led to creation of the Task Force, the broad representation of its members, as well as its mandate and goals. Subsequently it outlines the Task Force’s progression through three distinct phases, each marked by extensive consultation and culminating in a comprehensive report submitted to Health Canada. A particular focus is placed on the third phase, which resulted in the formulation of *An Action Plan for Pain in Canada*, and we present an overview of the recommendations contained therein. Moreover, the article situates the Canadian Pain Task Force within the broader movement to transform how pain is recognized, understood, and treated in Canada. It highlights initial steps taken to address identified priorities, indicating a proactive approach toward effecting meaningful change.

## RÉSUMÉ

En 2019, Santé Canada a créé le Groupe de travail canadien sur la douleur. Par cet engagement, le Canada s’est joint à d'autres pays, comme les États-Unis et l'Australie, pour mettre en place un mécanisme national visant à soutenir le travail dans le domaine de la douleur chronique. Cet article présente un historique des efforts de plaidoyer nationaux et régionaux qui ont conduit à la création du Groupe de travail, la représentation diversifiée de ses membres, ainsi que son mandat et ses objectifs. Il décrit ensuite la progression du Groupe de travail en trois phases distinctes, chacune marquée par de vastes consultations et aboutissant à la présentation d'un rapport complet à Santé Canada. Une attention particulière est accordée à la troisième phase, qui a abouti à la formulation d'un Plan d'action pour la douleur au Canada. Nous présentons également un aperçu des recommandations qu'il contient. En outre, l'article situe le Groupe de travail canadien sur la douleur dans le cadre d'un mouvement plus large visant à transformer la façon dont la douleur est reconnue, comprise et traitée au Canada. Il met en lumière les premières mesures prises pour répondre aux priorités recensées, ce qui témoigne d'une approche proactive visant à apporter des changements significatifs.

## Introduction

Chronic pain is often an invisible health condition that can have a significant impact on the physical, spiritual, social, mental, and economic well-being of the individuals living with it, as well as their families. Chronic pain may be secondary to other health conditions, such as diabetic neuropathy and arthritis, or exist irrespective of other health conditions (e.g., fibromyalgia). In 2019, the World Health Organization recognized this in its 11th iteration of the *International Classification of Diseases* by classifying chronic primary pain as a disease in its own right.^1^ Globally, chronic pain–related conditions, such as low back pain and headaches, are among the top causes of disability-adjusted life-years (DALYs).^2^ In Canada, it is estimated that nearly 8 million or one in five individuals, across the lifespan, live with chronic pain. Furthermore, “chronic pain disproportionately affects seniors, people living in poverty, people living with mental health and substance use disorders, people working in the trades and transportation industry, Veterans, Indigenous Peoples, certain ethnic and racialized communities, sexually and gender diverse persons, those who have experienced past trauma or violence, persons with disabilities, and women.”^3(p3)^ Chronic pain is also the most common disability type reported by Canadians.^4^ Despite significant impacts, the level of awareness, knowledge, and care for chronic pain among health care providers and the general public in Canada is often inadequate,^5^ with correspondingly low investments in pain research, surveillance, and other enabling supports for improving outcomes.

In 2019, the Government of Canada, under then–Minister of Health Ginette Petitpas Taylor, established the Canadian Pain Task Force (CPTF) to provide advice to Health Canada regarding evidence and best practices for the prevention, diagnosis, and management of chronic pain. This article provides a historical narrative of the CPTF, its goals and mandate, the many inputs into its work, as well as early results and actions aligned with its final report, *An Action Plan for Pain in Canada*. It situates the CPTF in the broader movement to transform how pain is recognized, understood, and treated in Canada and outlines early advocacy, education, and research that laid the foundation for the CPTF. It articulates how the opioid overdose crisis drew further attention to the significant impacts of chronic pain and spurred the establishment of the CPTF, among other actions.

This narrative accounts for chronic pain through the lens of the CPTF, its activities, and members. As a result, it does not include the multitude of experiences, perspectives, and activities of other organizations, individuals, and initiatives involved in this space. With this in mind, the authors would like to acknowledge and thank everyone who contributed to the work of the CPTF and the development of the *Action Plan*, in particular people living with pain who have been engaged throughout this process.

## Everywhere and nowhere: the early landscape of chronic pain in canada

In establishing the CPTF, Canada joined several other countries, notably Australia^6^ and the United States,^7^ in providing a national mechanism to support work in the area of chronic pain. The momentum that resulted in the CPTF being established in 2019 began more than two decades earlier, with advocates, researchers, and a loose network of specialist clinicians working on the issue of pain. Though the Canadian Pain Society (CPS) began its activities in 1975, the early 2000s witnessed significant momentum across Canada. People living with arthritis in Canada started making use of the internet to connect, share resources, and advocate for those living with the condition. In 2001, the Canadian Arthritis Patient Alliance was officially launched by roughly 80 arthritis advocates to “make arthritis better known, remove its devastating effects and create better lives,”^8(p3)^ a foreshadowing of the CPTF’s chronic pain-related objectives that would come nearly two decades later. The Canadian Arthritis Patient Alliance represented one of the first virtual, patient-driven organizations in Canada that included pain as a priority. They were closely followed by the no-longer-active Canadian Pain Coalition in 2003 and the Association québécoise de la douleur chronique in 2004, whose mission is still to improve the condition of people living with chronic pain in Quebec and reduce their isolation.

In 2010, more than a decade prior to the release of the CPTF *Action Plan*, the International Association for the Study of Pain held its first International Pain Summit in Montreal. One of the outcomes of this Summit was the Declaration of Montreal: “that access to pain management is a fundamental human right.”^9^ The Declaration further noted that inadequate pain management is largely a global issue.^9^ In a similar vein, in 2011 Dr. Mary E. Lynch, then-president of the CPS, highlighted the need for a Canadian pain strategy, noting that pain (both chronic and acute) is poorly managed and understood due to underrecognition and a lack of education and training.^5^ Later in 2011, this observation evolved into a more substantive document (unpublished), *Call to Action: The Need for a National Pain Strategy for Canada*, which called upon federal, provincial, and territorial governments to develop a coordinated approach to pain in Canada. Developed by the CPS and the Canadian Pain Coalition, it focused on access to care, awareness and education, research, and ongoing monitoring for pain. Between this early advocacy work and the establishment of the CPTF, several other initiatives also sought to articulate why and how chronic pain should be prevented and treated in Canada. These included the 2012 CPS Pain Summit, the 2016 Canadian Institutes of Health Research Canadian Pain Research Summit,^10^ the 2017 McMaster Health Forum Stakeholder Dialogue,^11^ the 2017 Arthritis Society Pain Symposium,^12^ and the 2018 Canadian Academy of Health Sciences Major Forum on Chronic Pain.^13^ Building on this momentum, additional nongovernmental organizations (Pain BC in 2008, People in Pain Network in 2012, SaskPain in 2018, among others) and national research and knowledge mobilization networks (Chronic Pain Network in 2016, Solutions for Kids in Pain in 2019, the Center of Excellence for Chronic Pain in Canadian Veterans in 2019) were created. Despite these efforts, recognizing chronic pain as a Canadian health priority remained a challenge.

In the mid-2010s, North America was impacted by a significant rise in opioid-related harms and deaths. Recognizing the potential role of the rapid increases in opioid prescribing in the early 2000s on the overdose crisis, policymakers began to implement efforts to curtail the supply of prescription opioids. At the same time, emerging guidance documents were being published in Canada and the United States, which questioned the effectiveness of opioids as a treatment for chronic pain. Though these actions were well intentioned and had positive impacts for some people (e.g., avoidance of unnecessary exposure to opioids and associated harms, successful opioid dose reduction leading to improved pain, etc.), they also had significant unintended consequences for many Canadians living with chronic pain. Some Canadians have since had difficulties accessing their opioid medications to help manage their pain, and others have had their opioid dose rapidly tapered or discontinued altogether.^14^ Lack of access to pain medications has also led some individuals to turn to illicit opioids to manage severe pain, increasing the risk of overdose or death.^15^ Emerging evidence also suggests that some individuals living with pain have sought medical assistance in dying services due to inadequate control of pain or concern about controlling pain.^16^ Increased stigma, anxiety, and fear surrounding opioid use have compounded these challenges and created additional barriers for people living with pain in accessing opioid medications to manage their condition.^15^

Recognizing these challenges, Health Canada engaged with pain experts, people living with pain, researchers, and pain specialists to better understand and identify ways to address the situation. These engagements culminated in a roundtable discussion with these key partners with then–federal Minister of Health the Honorable Ginette Petitpas Taylor, as well as targeted pain sessions included at the Opioid Symposium held in September 2018. Participants were able to share the challenges they had experienced, particularly those related to stigma and inconsistent access to treatment, as well as the ways in which responses to the overdose crisis, such as prescribing changes, exacerbated these challenges.^17^ The 2018 symposium represented the first time there were concurrent sessions on chronic pain with broad representation from the community. In her closing remarks at the symposium, Minister Petitpas Taylor committed to “exploring the establishment of a Pain Task Force.”^18^ On April 3, 2019, Minister Petitpas Taylor formally announced the creation of the CPTF during opening remarks at the Canadian Pain Society’s 40th Annual Scientific Meeting in Toronto.^18^

## Creating the Canadian Pain Task Force

Creating the CPTF led to a national-level body mandated to provide information to the federal government to help understand and address the needs of Canadians living with pain. Though the 2018 opioid symposium and the impact of the overdose crisis on people living with pain was the genesis behind the minister’s commitment to creating the CPTF, the federal government recognized that the Task Force’s mandate and terms of reference needed to identify gaps and best practices for the prevention and management of chronic pain more broadly; opioids were only one component of the broader issue. As such, the mandate was divided into three specific phases, with an associated report for each. The primary audience for the three reports was Health Canada and the secondary audience was the broader public, with particular relevance for people who live with pain and advocates and other key partners participating in activities related to pain management (e.g., researchers, health professionals, government officials, community organizations, and professional associations).

The process for drafting the mandate of the CPTF and for selecting its members was guided by Health Canada’s Policy on External Advisory Bodies.^19^ The membership selection process attempted to balance and provide representation from various perspectives, including those of people with lived experience, pain researchers, and experts providing health services to Canadians living with chronic pain. It also reflected the need for diverse and inclusive membership, reflecting the views of specific populations (e.g., Indigenous populations), gender, official language communities, and geographic locations. At the same time, there was a recognition that pain impacts multiple populations and spheres of life and its treatment does not belong to only one discipline or clinical practice. Following the selection of the Task Force members, gaps remained in the expertise required to identify evidence-informed priority actions to address a very complex health issue. The establishment of an Expert Advisory Panel^20^ (EAP) to support the work of the Task Force was meant to address the gaps in some areas, though some remained, including allied health (e.g., occupational therapy) as well as certain medical specialties.

In addition to the EAP, the Task Force was supported by several governance structures, including direct support from the Canadian Pain Task Force Secretariat (housed within Health Canada). The Task Force also received indirect support though the Federal Working Group on Pain and the Federal, Provincial, and Territorial Working Group on Pain, both of which were convened and led by the Secretariat. The working groups brought together federal, provincial, and territorial representatives whose departments, files, and/or mandates touched on the issue of chronic pain. Established after the launch of the first report, these working groups provided an opportunity for members to learn about Task Force activities and findings, as well as feeding into activities (such as the regional workshops).

The Task Force members were optimistic that for the first time there existed a formal commitment by the Government of Canada to potentially address the needs of people living with pain. This was seen as an opportunity to build on work already underway across the country—in peer support groups, clinics, research labs, classrooms, and beyond. They saw it as a platform to raise the voices of people impacted by pain, to prompt action by all levels of government, and to lay the foundation for a national pain strategy, which would lead to tangible benefits for Canadians affected by pain, their families, and society.

## Mandate of the Canadian Pain Task Force

The CPTF was tasked with providing advice to Health Canada regarding evidence and best practices for the prevention and management of chronic pain. This work was achieved through three distinct phases, each marked by extensive consultation and culminating in a comprehensive report submitted to Health Canada (Table 1).

**3a. Phase I—Assessing current state of chronic pain in Canada (leading to the first report: *Chronic pain in Canada: Laying a foundation for action*)**

**Table 1.** Phases of the mandate of the Canadian Pain Task Force.

Phases	Reports
Phase I: Assess how chronic pain is currently addressed in Canada	*Chronic Pain in Canada: Laying a Foundation for Action* (June 2019)
Phase II: Conduct national consultations and review available evidence to identify best and leading practices, potential areas for improvement, and elements of an improved approach to the prevention and management of chronic pain in Canada	*Working Together to Better Understand, Prevent, and Manage Chronic Pain: What We Heard* (October 2020)
Phase III: Provide recommendations on priority actions to ensure that people with pain are recognized and supported and that pain is understood, prevented, and effectively treated across Canada	*An Action Plan for Pain in Canada* (March 2021)

Phase I of the CPTF sought to provide an accessible overview of the current state of chronic pain in Canada, with the following objectives: defining and building literacy around pain, its prevention, and management; outlining the impacts pain has on individuals, families, and society and reflecting on the rationale for why actions are needed; providing a high-level description of chronic pain activities across Canada, including strengths, weaknesses, opportunities, and threats as well as broad evidence gaps; and, finally, articulating the main areas of inquiry that would eventually guide Phase II of the CPTF mandate. The Task Force was provided with a very ambitious timeline to release its first report reflecting on the evidence gathered by June 2019—only 4 months following Task Force creation.

To provide this in-depth overview of the state of chronic pain in Canada, activities during this phase and the writing of the report took a collaborative and iterative approach between the CPTF, the EAP, and the Task Force Secretariat. Activities conducted during this first phase included consulting academic and medical publications, gathering the professional experience of CPTF and EAP members, reviewing materials internal to Health Canada, as well as reviewing prior reports from key pain organizations and initiatives (such as those mentioned in the report’s introduction). Considering the multiple perspectives and spheres of expertise on the CPTF and EAP, this allowed for a relatively comprehensive overview. Though the first phase of the mandate produced a substantial amount of information, the authors avoided an overly clinical approach to make the report accessible to a diverse audience. The Secretariat also worked with the CPTF to identify Canadians willing to tell their story of living with chronic pain, with a focus on the barriers faced, treatments, and resources accessed, as well as thoughts on the mandate of the Task Force in general. The stories from this consultation were interspersed throughout the report and helped ground and humanize the issue of chronic pain.

As noted in the introductory section, several partners and reports have analyzed the state of pain treatment in Canada, highlighting its inadequacy. Though the Phase I report included some similar challenges, it balanced this with current strengths and resulted in a number of findings that were relevant to Health Canada and other pain partners, particularly: (1) the strength of education and research in Canada; (2) examples of innovative and promising practices; (3) existing barriers; (4) factors that render many treatment options largely inaccessible, including limited access to timely and appropriate multimodal care; (5) significant gaps in chronic pain surveillance and health system quality monitoring, education, training, and awareness for individuals and health care professionals and research and related infrastructure; (6) Canada’s internationally renowned research, as well as the lack of translation of breakthrough science into practice; and (7) education of people living with pain and those who care for them is limited, despite evidence that education is one of the most impactful levers for change. The Phase I report also articulated the impact of the opioid overdose crisis and increased challenges faced by people who require opioids to manage their pain and provided the evidence base to address these challenges and better support these individuals. It also explored the intersections between chronic pain, mental health, and substance use. In setting the context for the subsequent reports, Phase I framed pain in a holistic way by bridging the divide between physical manifestations of pain and mental and emotional trauma, in addition to exploring the range of social and cultural determinants that might influence the experience of pain.

**3b. Phase II—Conducting national consultations (leading to the second report: *Working together to better understand, prevent, and manage chronic pain: What we heard*)**

Building on Phase I, Phase II of the Task Force mandate focused on identifying areas for improvement and assessing options that would enhance the prevention and management of chronic pain in Canada. Though people with lived experience of pain were consulted during Phase I, consultations were expanded for Phase II and included national consultations with key partners—pain organizations and networks; federal, provincial, and territorial governments; health professionals; researchers; people living with pain; and specific populations (e.g., Indigenous Peoples). Unlike Phase I, Phase II intended to include active in-person options such as bilateral meetings, key informant interviews, focus groups, regional roundtables, and roundtables with targeted groups. There were also more passive options, such as a guided online questionnaire, solicitation of personal stories, and public opinion research or surveys. Within the CPTF and Secretariat, activities included a review of available evidence in Canada and internationally and oral source interviews with specific researchers or organizations, such as McMaster Health Forum, Cochrane Response, etc. The Secretariat also worked with federal partners to gather additional evidence. This included, for instance, a Canada’s Drug and Health Technology Agency rapid review, Drug Safety and Effectiveness Network Methods and Applications Group for Indirect Comparisons rapid review, and the Canadian Center on Substance Use and Addiction.

Phase II was a significant opportunity for engagement, and the report represented the voices of nearly 2000 people who were able to share their experiences through in-person, written, and online consultations. Several successful in-person consultations took place in the latter months of 2019 and into early 2020, including regional workshops in Nova Scotia, Quebec, Ontario, and Alberta, as well as a particularly compelling Indigenous consultation workshop that was undertaken as a Talking Circle at the Canadian Museum of Human Rights in Manitoba. Due to the global COVID-19 pandemic, like many in-person events during this time, planned consultations were delayed, switched to virtual, or canceled outright—including the in-person workshop that was planned for the Northwest Territories in April 2020. Despite these unexpected challenges, the Task Force was able to pursue national consultations virtually, achieving its mandate and delivering its second report on the findings of its national consultation in October 2020.

In reflecting on Phase II, the Task Force members were grateful to have been able to document the evidence, ideas, stories, and practices they heard through their extensive national consultation—despite much of this taking place in the context of two public health crises, the COVID-19 pandemic and record high numbers of opioid overdose deaths. It was a privilege for members to hear from people across Canada through a series of engagement activities to identify best practices and elements of an improved approach to pain care, education, research, and data in Canada. In their view, the engagement process not only brought forward best, promising, and emerging practices but it contributed to the mobilization of a network of people who live with, and care about, pain in Canada. In addition to altering the structure, and sometimes the ability to hold consultations at all, the COVID-19 pandemic also cast a harsh light on the accessibility and availability of treatments for pain, substance use, and mental health, particularly in cases where all three intersect.

**3c. Phase III—Identifying priority actions (leading to the third and final report: *An action plan for pain in Canada*)**

The final phase of the Task Force’s mandate was initially planned to take place between October 2020 and December 2021. The original focus for this final phase was to collaborate with key partners, including the chronic pain community; federal, provincial, and territorial governments; health professionals; researchers; and Indigenous populations, to disseminate information gathered over the previous phases related to best practices for the prevention and management of chronic pain. The Task Force was to then deliver a final report summarizing key activities undertaken over its 3-year mandate, with an update on progress related to the state of chronic pain management in Canada, by December 2021.

After completion of its second report, CPTF members wrote to then–federal Minister of Health Patty Hajdu and recommended that additional action be taken to address untreated pain in Canada. CPTF members were open to discussing adjustments to the Task Force mandate to better leverage their expertise to meet Health Canada’s and the minister’s priorities and pressures resulting from the pandemic. In response, and recognizing the significant challenges reflected over the course of the first two phases of the mandate taken together with the impact of a number of public health challenges for people living with pain, Health Canada revised and empowered the Task Force mandate in two ways: firstly by seeking their formal advice, through the provision of recommendations on priority actions, so that people with pain are recognized and supported and that pain is understood, prevented, and effectively treated across Canada, and, secondly, by accelerating the timeline for this empowered mandate by 9 months with a revised due date of March 2021 for the Task Force to deliver its final report.

Prior to drafting the final report, there was an extensive process to review evidence generated through the first two reports, as well as reviewing national health strategies developed in Canada since the year 2000 and national pain strategies developed in other countries. The CPTF then mapped these findings against health and social priorities for Canada to define overarching goals (Table 2) and specific recommendations. As with the report drafts in prior phases, there was a significant iterative process with feedback from the CPTF Secretariat, Task Force members, and the EAP. Based on these findings, the first five priority goals reflected in the final report fell fairly naturally into place. Though improved access to pain care was initially considered an obvious contender for the first priority, it was realized that without coordination, collaboration, and leadership across Canada, progress in *any* area may be impossible. As such, this was moved up to be the first goal in the report. The following two goals—improving access to pain care and better education, knowledge, and skills in pain assessment and management—were uncontentious. The fourth and fifth priorities became research, knowledge translation, and surveillance—recognizing the importance of better understanding and monitoring the burden of pain in Canada, as well as addressing inequities in how people across Canada fare when it comes to pain care. The area that received the most discussion was whether the equity lens should be integrated into each section or exist as a stand-alone section. Given the current attention to equity across Canada and the impact of inequity on people who live with pain, the Task Force reached a consensus for this to become a stand-alone goal as part of its final report.

**Table 2.** Overarching goals of *An Action Plan for Pain in Canada*.

**Goal #1**: Pain is recognized as a public health priority, and coordination of action across jurisdictions spurs collaboration, leadership, and support to ensure a consistent approach to pain throughout Canada
**Goal #2**: People have equitable and consistent access to a continuum of timely, evidence-informed, and person-centered pain care and supports across jurisdiction
**Goal #3**: People living with pain and health professionals have the knowledge, skills, and educational supports to appropriately assess and manage pain based on population needs. The broader community understands pain as a legitimate biopsychosocial condition and stigma is reduced
**Goal #4**: Pain research and related infrastructure enable discovery, catalyze innovation, and result in the translation of knowledge into real-world impact
**Goal #5**: Data enable effective monitoring of pain and facilitate improvement of health system quality
**Goal #6**: There is improved and equitable access to services for populations disproportionately impacted by pain

Recognizing the broad scope of the recommendations, the Task Force highlighted the following actions in its transmittal letter to the minister as a way to aid the prioritization of next steps for Health Canada: (1) enable collaboration and coordination as a necessary foundation for achieving the full suite of goals by convening relevant partners with a role to play in the *Action Plan*’s implementation to discuss joint commitments to action; (2) address the critical lack of access to pain care by establishing a funding mechanism to enable the development and coordination of community-based pain initiatives and issuing a statement to end discrimination against people living with pain and denial of care based on a history or current use of opioids; (3) help build awareness and understanding around pain as a disease that is invisible and often stigmatized by developing and implementing a pan-Canadian public awareness campaign to help communicate ideas around how pain works, risk, and protective factors and to connect people with needed resources and supports; (4) drive innovation, discovery, and knowledge creation and mobilization by establishing pain as a cross-cutting priority at Canada’s research funding agencies; and (5) enable national pain indicators and surveillance initiatives across jurisdictions by convening federal, provincial, territorial, and local governments; data stewards; clinicians; and people living with pain to discuss how best to collaborate.

With exactly a decade between the 2011 *Call to Action: The Need for a National Pain Strategy for Canada* and the 2021 *An Action Plan for Pain in Canada*, the Task Force’s final report represented a significant step toward articulating concrete actions that can be taken to improve the state of pain care across the country.

## Discussion: early wins and progress in implementing *an action plan for pain in Canada*

Though the CPTF mandate officially ended on December 31, 2021, work has continued at multiple levels, including within the federal government and within the Canadian pain community, to support effective implementation of *An Action Plan for Pain in Canada*. For instance, a dedicated policy team was established within Health Canada to support and coordinate federal efforts on chronic pain, including implementing priority actions identified by the Task Force. The team is working collaboratively with federal representatives in several government departments to advance recommendations and identify federal opportunities to support pain priorities. At the community level, pain organizations and people living with pain have come together to establish Pain Canada—a national action network to coordinate efforts and mobilize resources and tools dedicated to improving health outcomes of people living with pain. These two entities are building on the relationships established through the CPTF, utilizing both top-down and bottom-up strategies, to support the implementation of priority actions identified by the Task Force.

Members of the pain community also leveraged new opportunities to raise awareness and to undertake new initiatives or renew or expand existing ones to implement some of the Task Force’s recommendations. The following are examples of key national-level initiatives undertaken since the release of the Task Force’s final report: (1) existing federal grants and contributions programs (e.g., Health Canada’s Substance Use and Addictions Program, Health Care Strategies and Policies Program) were leveraged to support projects addressing pain priorities reflected in the Task Force final report; (2) the Chronic Pain Network mandate was renewed for an additional 4 years with a focus on knowledge mobilization and implementation science; (3) the Power Over Pain Portal was launched—a national virtual platform dedicated to provide online resources for youth and adults living with pain; (4) Solutions for Kids in Pain, in partnership with the Health Standards Organization, created and published the *Pediatric Pain Management Standard*—a first of its kind in the world; (5) New pain organizations have been created—including Pain Ontario—to foster community capacity to address pain; and (6) Pain Canada has scaled several education and self-management programs nationally and launched the “Action Tracker” to monitor implementation activities.

## Conclusion/the way forward

Though progress has been made over the past 3 years since publication of the *Action Plan*, significant work remains to effectively address priority actions identified by the CPTF and for pain to be reflected as a national health priority. As noted in the CPTF final report, a range of actors working inside and outside of the health system, including all levels of governments, peer advocates, nongovernmental organizations, academic institutions, insurers, and others, must step forward to enable positive and lasting change. Efforts need to continue to bring awareness around the significant impacts of chronic pain, to promote progress, and to reflect on remaining gaps, examples of which are included in Table 3.

**Table 3.** Notable gaps in the implementation of *An Action Plan for Pain in Canada*.

Overarching Goals	Notable Gaps
Goal #1	Creating a funding mechanism for federal/provincial/territorial and community-based initiatives, with emphasis on supporting patient- and peer-led organizations
Goal #2	Advancing incentives to improve pain assessment and management across the continuum of care, including policy enablers, standards, remuneration, and others
Goal #3	Creating a public awareness campaign to increase pain literacy
Goal #4	Investing in pain research infrastructure and increasing support for both discovery and mobilizing knowledge
Goal #5	Ensuring national pain surveillance to better describe the burden of pain and the disparities that exist across different populations
Goal #6	Reducing disparities in access to pain care for equity-deserving populations and across geographies

Opportunities exist to address these gaps and continue the considerable momentum that is underway since the closeout of the CPTF. For instance, in April 2024, CPS, Health Canada, and Pain Canada co-organized a National Pain Congress in the margins of the 2024 CPS Annual Scientific Meeting. This represented an opportunity to foster new collaborative relationships and knowledge exchange between many different actors, including not only patient partners, health professionals, and researchers but also, importantly, policymakers across multiple federal agencies and departments and provincial/territorial representatives, with the common goal of improving health outcomes for people affected by pain. Opportunities must be leveraged to expand engagement of partners across different sectors, domains, experiences, and expertise to move us toward full implementation of *An Action Plan for Pain in Canada*.
